# COVID-19 pandemic lockdown responses from an emotional perspective: Family function as a differential pattern

**DOI:** 10.1093/geroni/igaa057.3437

**Published:** 2020-12-16

**Authors:** Javier Lopez, Gema Perez-Rojo, Cristina Noriega, Cristina Velasco, Isabel Carretero, Patricia López-Frutos, Leyre Galarraga

**Affiliations:** 1 Universidad San Pablo CEU, Boadilla del Monte, Spain; 2 Universidad San Pablo-CEU, Alcorcón, Spain

## Abstract

Family can be an essential resource at times of loss or vital crisis. Loneliness and isolation in older adults might have serious negative consequences for their mental health. For this reason, this research aims to analyze the role of family function in the anxiety and depression experienced by older adults during the COVID-19 crisis. Participants were 882 Spanish community-dwelling adults over 60 years of age. Sociodemographic characteristics, characteristics related to the coronavirus, self-perceived health, family function, avoidance, depression and anxiety were analyzed. Data suggest a buffering effect of family function on anxiety and depression during the pandemic. Furthermore, being unmarried or a female, greater fear of COVID-19, worse self-perceived health, greater avoidance, and worse family function were associated with higher levels of anxiety. Likewise, greater fear of COVID-19, poorer self-perceived health, greater avoidance, and poorer family function, were associated with greater depression. These results point out that family dysfunction is a predisposing factor for the development of the emotional problems of anxiety and depression in older people in potentially stressful and loss situations.

